# Editorial: Immunometabolic alterations linked to affective disorders and other mental illnesses

**DOI:** 10.3389/fpsyg.2025.1699733

**Published:** 2025-10-02

**Authors:** Yolanda Sánchez-Carro, Pilar López-García, Alejandro de la Torre-Luque

**Affiliations:** ^1^Department of Psychology, Madrid Open University (UDIMA), Madrid, Spain; ^2^Center for Biomedical Research Network in Mental Health (CIBERSAM) Carlos III Health Institute (ISCIII), Madrid, Spain; ^3^Department of Psychiatry, Universidad Autonoma de Madrid (UAM), Madrid, Spain; ^4^Department of Legal Medicine, Psychiatry and Pathology, School of Medicine Universidad Complutense de Madrid, Madrid, Spain

**Keywords:** inflammation, metabolic syndrome, mental disorders, affective disorders (other), anxiety disorders (other), schizophrenia

## Introduction

The relationship between the immune response and mental health has shifted from being an emerging hypothesis to becoming a central focus in contemporary psychiatric research. The immune system, whose primary function is to defend the organism and maintain homeostasis, responds adaptively to infections or tissue damage. However, when this response becomes dysregulated, it can turn pathological and contribute to the development of a range of diseases. Today, we know that environmental factors that may lead to chronic stress, traumatic events, or prolonged adversity can activate inflammatory pathways even in the absence of external agents. This phenomenon has opened a field of research: the role of immunometabolic alterations in mental disorders.

Over the past decades, depression has become the mental disorder that probably best illustrates this relationship. Clinical, epidemiological, and experimental studies have demonstrated associations between systemic and brain inflammation and depressive symptoms, and specific immunological profiles have been described according to diverse symptom profiles. These findings have fueled an intensive search for biomarkers that may improve diagnostic stratification, risk prediction, and the development of personalized interventions. However, many questions remain unanswered.

This Research Topic gathers evidence from four original articles that, from different methodological perspectives, deepen our understanding of this research area within the framework of translational psychiatry.

## Systemic inflammation as an early marker in psychiatric disorders

The search for accessible and reliable biomarkers capable of accurately differentiating psychiatric disorders from their early or prodromal stages remains one of the great challenges of current psychiatry. Research has mainly focused on hematological and lipid indicators, as they are easily obtained through routine analyses, inexpensive, and widely applicable in clinical settings. Nevertheless, other non-invasive sources, such as saliva, are also being explored and may provide complementary and valuable information. Identifying differential inflammatory profiles is relevant not only diagnostically but also prognostically and therapeutically.

In this line, Qiu et al. evaluated the diagnostic potential of eight inflammatory biomarkers derived from hematological and lipid parameters in a cohort of more than 600 individuals, including patients with first-episode schizophrenia, bipolar disorder, depression, and healthy controls. Among the indices analyzed, four stood out: the neutrophil-to-lymphocyte ratio (NLR), the systemic immune-inflammation index (SII), the monocyte-to-HDL ratio (MHR), and the neutrophil-to-HDL ratio (NHR), providing evidence for the involvement of immune-inflammatory reactions and dyslipidemia in the development of these psychiatric disorders. Moreover, their results underscore the superior predictive power of combined biomarker assessments for diagnostic classification [e.g., NHR + MHR + NLR achieved areas under the curve (AUC) >0.80 for distinguishing schizophrenia from controls while in depression the NHR + MHR combination achieved an AUC of 0.82].

This study underscores the potential of accessible, cost-effective biomarkers derived from routine analyses as diagnostic support tools in clinical practice. Furthermore, it provides evidence of differentiated inflammatory and dyslipidemia profiles among psychiatric disorders, reinforcing the idea of biological heterogeneity within each diagnostic category.

## Low-grade inflammation and depression in population studies

Population-based studies are essential to validate findings observed in smaller clinical cohorts. High-sensitivity C-reactive protein (hs-CRP), a classic marker of low-grade inflammation, has been extensively investigated in relation to depression, though results have been heterogeneous. Examining its association in large, representative samples helps establish more robust patterns and explore modulators such as sex or age.

Ji et al. presented a highly representative population-based study. Using NHANES 2017–2020 data (6,293 U.S. adults), they examined the association between hs-CRP levels and the presence of depressive symptoms. The findings revealed a robust positive relationship: each unit increase in hs-CRP was associated with a 10% increase in the likelihood of depression, even after adjusting for multiple sociodemographic and clinical factors.

Additionally, participants in the highest hs-CRP quartile had a 39% higher likelihood of depressive symptoms compared to those in the lowest quartile. A particularly relevant aspect was the identification of sex differences: the association was stronger in men, while it was attenuated in women. These results reinforced the idea that low-grade inflammation constituted a risk factor for depression and highlighted the need to consider gender variables in future studies.

## Viral infections, neurophysiology, and inflammation in depression

The study of interactions between viral infections, inflammation, and neurophysiology has been an area of great interest in recent decades. Various viruses have been implicated as possible modulators of brain function and risk factors for developing affective disorders, although evidence has often been contradictory. Simultaneously examining neurophysiological and molecular markers is crucial to clarify whether infectious agents play a causal role in depression or if their influence is marginal.

Torner et al. approached this Research Topic in a cohort of patients with MDD and healthy controls. They analyzed the presence of Borna disease virus (BoDV-1), resting EEG activity, and levels of IL-6 and IL-8. The results revealed a characteristic EEG pattern in depression, with inversion of the alpha gradient between frontal and posterior regions, but no evidence of relevant effects of BoDV-1 on brain activity or cytokine levels.

Interestingly, a negative correlation was observed between frontal alpha power and IL-8 levels in BoDV-1-negative depressed patients. This finding suggests a potential link between neurophysiological activity and inflammatory markers, although the absence of a significant role for Borna virus invites a rethinking of hypotheses regarding infectious factors in depression. This study illustrates how integrating neuroscience, immunology, and virology can broaden the spectrum of factors explored in affective disorders.

## Lipid metabolism and suicide risk in depression

Metabolic factors, particularly those related to lipid profiles, have attracted increasing interest in psychiatry. Lipids not only influence cardiovascular health but also play a key role in modulating immune and inflammatory responses, which in turn are involved in neurobiological processes linked to depression and suicidal behavior. Alterations in lipid metabolism may therefore contribute to both systemic inflammation and changes in brain function. Exploring these connections offers the possibility of identifying early biomarkers that enable more precise interventions.

Xu et al. explored for the first time the relationship between remnant cholesterol (RC) levels and suicide attempts in Chinese patients with a first episode of untreated major depression. In a large sample of 1 718 participants, they identified a suicide attempt prevalence of 20.1% and described a non-linear relationship between RC levels and suicide risk. Specifically, when RC was below 1.99 mmol/L, each unit increase was associated with a 36% higher likelihood of suicide attempt. Above this threshold, however, the association disappeared.

These results position remnant cholesterol as a potential emerging biomarker for suicide risk assessment, particularly in young patients. The finding is relevant not only because of the magnitude of suicide in major depression, but also because it links lipid biology with suicidal behavior through mechanisms such as inflammation, insulin resistance, endothelial dysfunction, or alterations in gut microbiota.

## Conclusion and future implications

The four studies included in this Research Topic highlight the richness and diversity of approaches in the study of immunometabolic alterations in mental health. From large-scale population studies to biomarker analyses in first episodes, investigations integrating neuroscience and immunology, and clinical cohorts with suicide risk—all converge on the same message: psychiatry cannot be dissociated from inflammatory and metabolic biology.

However, challenges also emerge. The heterogeneity of findings suggests that there is no single universal biomarker, but rather specific profiles in certain patient subgroups. These results reinforce the need to move toward precision psychiatry, combining biological, clinical, and sociodemographic markers to improve diagnosis, prevention, and intervention.

In this regard, the future of the field will enormously benefit from:

- Longitudinal studies to distinguish causal markers from epiphenomena.
- Integration of machine learning techniques to model complex and predictive patterns.
- Clinical trials incorporating anti-inflammatory therapies targeted at patient subgroups characterized by elevated biomarkers.
- A translational approach that connects molecular findings with practical interventions in psychiatric care.

This Research Topic invites to reflection and continued research at the intersection of immunology, metabolism, and mental health. The contributions presented here not only expand our scientific knowledge but also point to concrete pathways toward a biomarker-based, personalized medicine that could transform the approach to affective disorders and other mental health disorders in the coming years.

